# Global trends in the scientific research of the health economics: a bibliometric analysis from 1975 to 2022

**DOI:** 10.1186/s13561-023-00446-7

**Published:** 2023-05-12

**Authors:** Liliana Barbu

**Affiliations:** Faculty of Economic Sciences, Lucian Blaga University of Sibiu, 550024 Sibiu, Romania

**Keywords:** Health economics, Bibliometric analysis, CiteSpace, Scientometrics, I18

## Abstract

**Background:**

Health science is evolving extremely rapidly at worldwide level. There is a large volume of articles about health economics that are published each year. The main purpose of this research is to explore health economics in the world's scholarly literature based on a scient metric analysis to outline the evolution of research in the field.

**Method:**

The Web of Science repository was used to get the data (1975–2022). The study explores 1620 documents from health economics. CiteSpace software was used to provide network visualisations. Four thousand ninety-six authors, 1723 institutions, 847 journals and 82 countries were involved in the sample. The current research contains a descriptive analysis, a co-authorship analysis, a co-citation analysis, and a co-occurrence analysis in health economics.

**Results:**

Drummond M.F (author), the USA (country), University of London (institution) and Value Health (journal) are among the most important contributors to the health economics literature. Co-authorship analysis highlights that cooperation between authors, institutions and countries is weak. However, Drummond M.F. is the most collaborative author, the USA is the most collaborative country, and University of York is the most collaborative institution. The study offers an image about the most co-cited references (Arrow K.J., 1963), authors (Margolis H.) and journals (British Medical Journal). The current research hotspots in health economics are “behavioural economics” and “economic evaluation”. The main findings should be interpreted in accordance with the selection strategy used in this paper.

**Conclusion:**

All in all, the paper maps the literature on health economics and may be used for future research.

## Introduction

The health economy is a branch of the economy that deals with concerns of the production and consumption of health services and healthcare that relate to efficiency, effectiveness, value, and behaviour. Applying economic ideas, concepts, and methods to institutions, actors, and activities that have an impact on people's health is known as health economics [1]. The health economy is studying how to allocate limited resources to meet human desires in the medical industry and disease care. The health economy often tries to meet the most pressing challenges facing the health system. Studies in health economics provide to decision-makers precious information about the effective use of resources that are available to maximize health benefits.

The health economics is a component of public health, a component that It can be used to examine health issues and medical treatment. Health economists consider the origin of their discipline to Petty W. (1623–1687) [2] who propose valuation of human life based on a person’s contribution to national production. Arrow K. is credited with creating the field of health economics in a work where he conceptually distinguished between health and other goods [3]. Since Arrow K.'s fundamental publication on health economics from 1963, the scale of the healthcare sector, the share of public budgets allocated to healthcare, and the body of research on health economics have all increased significantly [4].

The current pandemic context has proved the need for a functioning public health system capable of meeting any challenges. The World Health Organization report for 2020 presents an examination of 190 nations' global health spending from 2000 to 2018. The report shows that global health spending has increased consistently between 2000 and 2018, reaching $ 8.3 trillion, or 10% of world GDP [5]. At the level of OECD Member States, the latest estimates show an average increase in health spending of about 3.3% in 2019, whereas health spending as a percentage of GDP stayed about where it had been in prior years, at 8.8% [6]. These indicators rose sharply in 2020, as economies faced a pandemic. The increases were driven by an increase in the level of allocation of government resources for health, while private spending on health tended to decline. At EU level, the public sector plays a major role in funding health services. In 2/3 of Member States, more than 70% of health spending is funded by the public sector [7]. In 2020, the EU's overall public health spending was €1.073 billion, or 8.0% of GDP (https://ec.europa.eu/eurostat/statistics-explained/index.php?title=Government_expenditure_on_health). For governments, public spending on health is one of the spending categories with the quickest growth.

Health economics is the application of economic theory, models, and empirical techniques to the analysis of decision-making by individuals, health care providers, and governments regarding health and health care. Even though the methodologies are distinct in terms of health care, health economics aims to apply the same analytical tools that would be applied to any good or service that the economy provides [8]. By offering a clear framework for decision-making based on the efficiency principle, health economics seeks to simplify decision-making [9]. Extensive government interference, insoluble uncertainty in many dimensions, asymmetric knowledge, barriers to entry, externality, and the presence of a third-party agent are all characteristics that set health economics apart from other fields [10].

Health economics is the field were interdisciplinarity bring additional value for society. Health economics development has not been without controversy. Health economics refers to a variety of elements that interact to affect the expenses and spending of the healthcare sector. Its controversy rises from the roles of people, healthcare providers, insurers, governmental bodies, and private companies in influencing the healthcare sector expenses. The parties that interact in this field have some conflicting goals. On the one hand, health care policymakers and public hospitals have as objective to provide real value to the patients, to balance public interest and economic restrictions. On the other hand, private hospitals, insurance companies aim to obtain profit for their shareholders. There are several weaknesses that should be rectified in the future. Among weaknesses it can be found deficiencies in the supply of health economists [11], a lack of financial resource independence between the local and central levels, the key macroeconomic variables' unfavourable behaviour, and the difficulty in developing new financing alternatives [12]. In addition to having too close relationships to national institutions and sponsors of health economics research, health economics also has excessively loose connections with general economic theory [13]. Considering increased demands in healthcare services and limited health care budgets, health economics faces real challenges in providing decision making frameworks and there will always be challenging healthcare decisions. Although it has not always been an impartial instrument, health economics does give useful information for policy [14]. Regarding how well economics integrates with promoting health, there is scepticism, and public health has mixed feelings on the subject. Health economics has been accused of focusing more on the consumption of healthcare services than the creation of healthcare [15]. Despite several methodological limitations, health economics can provide helpful concepts and principles that aid in comprehending the effects of resource allocation decisions [9]. All practitioners must have a elementary comprehension of some economic concepts to both understand the helpful ideas the field may provide and recognize its inadequacies.

The main purpose of the research is to examine the health economics literature published worldwide based on a scient metric analysis to outline the development of the field's research. The existence of a multitude of articles published on health economics determines the need to address and measure it quantitatively. Such an analysis is justified by the need must be aware of the current trends and future directions of research in the field of health economics. Health science is evolving extremely rapidly at worldwide level. There is a large volume of articles about health economics that are published each year. Another argument is that there several computer programs which allows for scient metric analysis of health economics publications. This article contributes to the bibliometric literature on health economics by offering answers to the subsequent research inquiries: How scientific production has evolved in health economics? Who are the most important authors and publications in health economics? What are the geographical and institutional hubs of knowledge production in health economics? What kind of collaboration between authors, organizations, and nations are there in the field of health economics research? Which are the most cited authors and the most cited papers, and which are the most attractive journals for publishing research results in health economics? What are the most debated conceptual approaches in health economics?

The remainder of the paper is structured as follows. The second section introduces a short literature review. Research methodology and data collection are presented in Sect. 3. Section 4 contains the quantitative and qualitative scient metric analysis on health economics by using CiteSpace software (descriptive analysis, collaboration analysis, co-citation analysis and keywords co-occurrence analysis). The last part concludes the analysis, presents the research limitations, and describes future directions of research.

## Literature background

Although there are thousands of articles published on health economics, very few articles aim for bibliometric analysis of the field and use computer programs. A first article published by Rubin, R. M. and Chang, C. F. (2003) aims at the study of 5,545 indexed articles, in the period 1991–2000, in the EconLit database, in the Health Economics section [16]. The second study is published by Wagstaff, A. and Culyer, A. J. in 2012 and extends the previous bibliometric research done by Rubin and Chang also based on the articles indexed in EconLit on health, over 40 years [17]. The third study, published by Moral-Munoz J.A et all in 2020, focuses on articles indexed in the Web of Science, between 2010 and 2019, which have the word "health" and do not use scientometric software [18].

It would be worth mentioning a descriptive analysis of the field conducted by Jakovljevic M. and Pejcic A. in 2017, but without the use of bibliometric indicators. The authors quantitatively analyze health economics publications by querying the PubMed, Scopus, WoS and NHS economic evaluation Database between 2000 and 2016 and conclude with the existence of an upward flow of health economics publications [19]. In this context, the proposed research is characterized by focusing on WoS articles that refer strictly to "health economics" and their computer processing to obtain maps and connections between studies.

## Research methodology

### Research methods

In the current paper two research methods were used: bibliometric analysis and knowledge mapping. Regarding the first one, it should be mentioned that bibliometric research methods are used delivering quantitative analysis of textual works, in this case publications about health economics. This method allows bibliographic overviews of scientific production in the field. In the scientific community, the technique is increasingly employed to provide details regarding relationships between various groups [20]. Bibliometric analysis uses statistical tools and different metrics as part of the analysis (frequency/ count, co-citation, co-authorship, co-occurrence, betweenness centrality, citation burst, modularity, centrality, sigma, Silhouette etc.). Bibliometric analysis naturally presents itself as a tool to qualify, then quantify, the study conducted [21].

Regarding the second one, bibliometric analysis uses a large quantity of information that should be transformed in knowledge. This is done by using data visualization and knowledge maps. An enormous and complex collection of knowledge resources can be more easily accessed and navigated by using knowledge mapping strategies [22]. Knowledge mapping is the process of making knowledge maps, it makes explicit knowledge graphic and visual. Knowledge maps are static, they are a “snapshot in time” that aids in understanding and organizing knowledge flow for researchers [23]. A process, method, or instrument called “knowledge mapping” is used to analyse knowledge to find traits or meanings and perceive knowledge in an understandable and transparent way [24]. One of the advantages of knowledge mapping includes the freedom to combine without restriction, i.e., without restrictions on the number of connections and concepts that can be established [25].

### Data source and search strategy

For this analysis we decided to use one of the most reliable databases: Web of Science (WoS) because it contains a data for large period. The data was retrieved from the Web of Science Core Collection by using title search tool TI = (health economics). The primary literature data were downloaded on 7th of October 2022. The query objective was to integrate in this analysis all research papers related to health economics. We did not introduce any restrictions regarding the topic or time span for searching documents. We intend to have a comprehensive view of the research area and to see its evolution over time. As a result, 2340 documents were retrieved. Among publications about health economics, the most numerous documents are the articles (37.6%), followed by editor materials (19.8%), meeting abstracts (13.9%) and book reviews (13.3%). There are also review articles on the subject, proceeding papers, letters, books, and book chapters which were kept in the sample. The other types of documents were removed resulting a sample of 2305 publications. The language of almost all publications is English (91.4%), followed by German (4.3%). The percentage of publications produced in other languages, such as French, Spanish, Portuguese, Russian etc. is less than 1.5% for each of them. Publications in other languages than English were eliminated, remaining 2108 documents in the sample.

The next step is to identify and remove duplicates by using Excel function (Conditional Formatting – Highlight Duplicate Values), therefore 8 duplicates were removed. In the sample under analysis, a multitude of types of documents indexed in WoS and referring to the concept of health economics can be observed. During the step of checking for duplications, it was found that there are too many duplicates of documents’ title, most of them due to editorial materials or book reviews. This led to a thorough analysis of publication by type of document (eg there are more than 10 reviews for one book or more than 10 editorial materials signed by the same editor). We identified some publications which are irrelevant for the purpose of our analysis. One hundred eighty-six editorial materials without citations and all 286 book reviews were removed resulting 1628 publications. We kept the editorial materials with citation because some of them have more than 100 citations. We searched for anonymous publications, more exactly we looked for incomplete data (author’s name is missing) and we removed 8 documents.

For the remaining documents the "Full Record and Cited References" was downloaded on 13th of October 2022 (txt files) and used as original data for the proposed bibliometrics analysis and science mapping. The final data collection, which consists of 1620 publications, is supported by 16,755 citing articles (excluding self-citations) and has been cited 18,504 times (excluding self-citations), giving it an H-index of 59. The data are statistical analysed by using annual distribution of publications, authors, journals. Co-authorship analysis focuses on collaboration between authors, institutions, and countries. Cited references, cited authors, and cited journal are used in co-citation analyses, and finally, the co-occurrence will integrate keyword in this research.

**Fig. 1:**
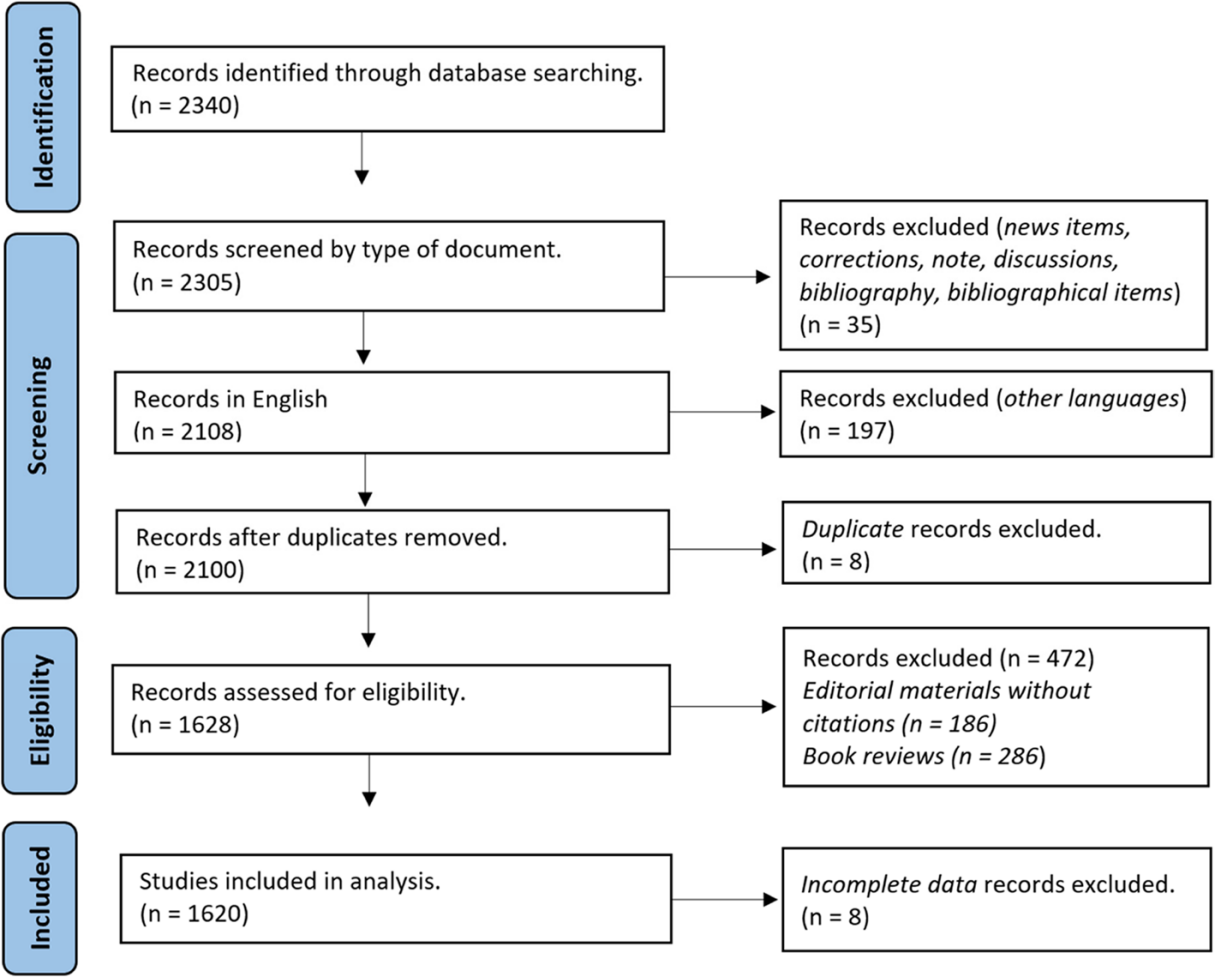
Selection procedure flow chart. Source: Authors

The graphical representation of selection procedure can be seen in Fig. 1.

### Visualization tools

Bibliometric method needs a certain amount of data to be statistically credible. This is the reason for that computerized data treatment is needed. Moreover, databases contain hundreds or thousands of entries which are analysed by using computer software. There is many bibliometric software, each of them has particularities and weaknesses. CiteSpace was chosen in this study because it is very user friendly, intuitively, and easy to use. CiteSpace 6.1.R2. available for free download at https://citespace.podia.com. A variety of networks created from scientific publications, such as collaboration networks, author co-citation networks, and document co-citation networks, are supported by structural and temporal analysis in CiteSpace. CiteSpace can produce knowledge domain X-rays. The CiteSpace parameters for this investigation were as follows: time-slicing was from 1975 to 2022, years per slice was 1 year, Look Back Years (LBY) = -1, Link Retaining Factor (LRF) = -1. For text processing and links, we preserved the default settings. We used several nodes (authors, institutions, journal, references, keywords) and metrics (such as citation burstiness, Sigma, Silhouette, rad Q, betweenness centrality) depending on the study that was done. Top N% is set to be equal to 100%, Top N is set to be 50, and g-index is set to be 25.

## Results

### Statistical analysis

**Fig. 2:**
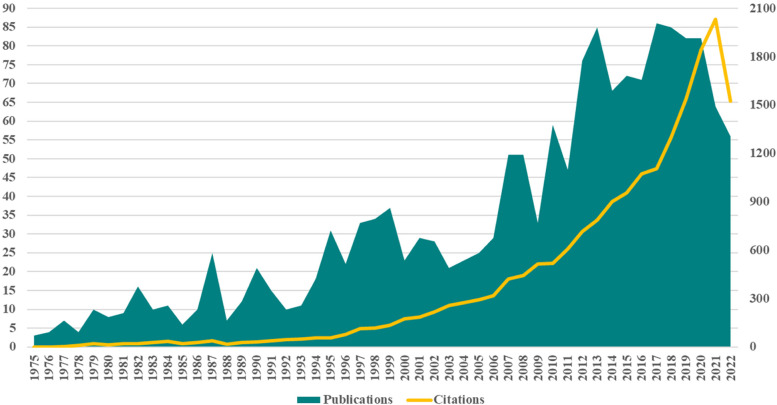
Literature production related to health economics between 1975 and 2022. Source: Authors

The first step to follow in the scient metric analysis is to analyse the evolution of publications’ number in the researched field. The way in which they are distributed over the years indicates the attention that the field of health economics has benefited from and the speed at which its conceptual development took place. The first 3 papers about health economics were published in 1975, indicating the lowest number of annual publications, but also a concept that has existed for over 4 decades. From Fig. 2, a general upward trend of health economics publications can be observed, but with numerous upward and downward fluctuations, generating sinusoidal cycles with an average duration of 3–4 years. The period 1975 – 1986 is characterized by a very low number of publications, 98 publications written by 110 authors in 12 years, representing 6% of the total sample. The next two decades (1987 – 2006) are characterized by a slightly increasing trend in the number of publications, with an annual average of approximately 23 publications on health economics, reaching a total of 454 publications written by 826 authors and representing 28% of the total number of analysed publications. Cyclical evolution is highlighted by booms in 1987, 1990, 1995, 1999, 2001.

The following period, 2007 – 2022 (16 years) is characterized by an upward evolution of the number of health economics publications, 1068 publications with an annual average of 67 articles (3261 authors involved), meaning 2.3 times more numerous as in the previous two decades and representing 66% of the total sample. In 2017, 86 studies on health economics were published, reaching the highest value in the analysed period. The quantitative evolution of publications in health economics it is explained by a higher interest of the researchers and policymakers to explore the benefits of health economics. The need to identify the ways in which health economics contributes to the healthcare system development represent a solid motivation to continue intensive research in the field.

The evolution of the citations’ number follows, like a shadow, the evolution of publications’ number. The upward trend is maintained, also respecting the previously presented temporal distribution, but without cyclical and sinusoidal fluctuations. The evolution of the citations’ number indicates the growing interest of specialists in researching the field, especially after 2000 when a constant and galloping annual increase in citations begins. The last 5 years show a very high interest of researchers and academics in health economics research, with a maximum point in 2021, with over 2000 citations, an evolution argued by the emergence of the global pandemic. All the figures and observations indicate a constant interest in the conceptualization of health economics and foresee a deeper development in the future.

Geographical analysis allows a better understanding of the field. The 1620 publications involved the work of authors from 82 countries. Among them, the first 10 states with significant contributions in the field of health economics stand out: the USA (605 papers), England (400), Canada (115), Australia (103), Netherlands (75), Scotland (64), Germany (59), Switzerland (57), France (47) and Italy (43). 96.8% of all publications were produced by top-10 countries. According to statistics, the USA is the top nation. 37% of all analysed documents are written by American authors, which is 1.5 times more than values recorded by England (rank 2) and 5.2 times more than Canada, rank 3. There are 49 nations where there are fewer than or equal to 5 publications during entire period.

In our study, a sum of 4096 different authors were identified, and they individually published between one and 16 papers, but only 170 persons are co-authors of more than 3 papers. Table 1 lists the top 10 authors with publications about health economics. Drummond M.F. is the leader, even if he published Essentials of Health Economics with his co-author, Mooney G.H., in 1982. He is affiliated to University of Yor (the UK). The top ten most productive authors published 107 articles, which represents 6.6% of the total publications. The most authors (95.8% of all authors) contributed to the health economics research with less than two papers. It should be noted that the number of authors is 2.5 times over the number of papers., which means that publications are made by cooperation between researchers.

**Table 1:** Top 10 most productive authors in health economics research

Rank	Authors	Pubs	%	h-index	Main focuses
1	Drummond M.F	16	0.98	51	Essentials of health economics, role of health economics, ISPOR special task force reports
2	Jonsson B	11	0.67	62	Health economics in cancer research, osteoporosis, vascular events, hypertension treatment
3	Coast J	10	0.61	43	Health economics: role, capability approach, qualitative methods, outcome measurement
4	Donaldson C	10	0.61	10	Coronavirus, resource allocations, health economics utility
5	Edwards R.T	10	0.61	32	Qualitative health economics, public health economics, micro-costing
6	Mooney G.H	10	0.61	22	Essentials of health economics
7	Neumann P.J	10	0.61	66	ISPOR special task force reports, costing methods, presidential candidates, and health economics
8	Palmer A.J	10	0.61	31	Health economics in osteoporosis, diabetes, bariatric surgery
9	Peeples P	10	0.61	2	Salaries in health economics
10	Postma M.J	10	0.61	58	Health economics of vaccines for different viruses

*Note*: Pubs = number of publications, % = share in total analysed documents (1620), h-index is based on the Web of Science Core Collection citations of the publications calculated for each author

Source: Authors

From the point of view of affiliation, the 4096 authors belong to 1723 institutions. The top 10 organizations with many health economics articles are University of London (91 publications), University of California System (54), University of York (51), Harvard University (45), University of Birmingham (41), University of Pennsylvania (34), University of Oxford (30), University of Aberdeen (28), University of California Los Angeles (28) and University of Washington (28). The list is dominated by institutions from the UK and the USA. The top-10 institutions contributed to health economics research field by 230 papers which represents 26.5% of total publications.

It is very important to see which journals have published the most articles about health economics. Regarding the publication’s titles, 847 distinct journals published all 1620 documents related to health economics. It should be mentioned that 782 journals (92.3%) published from one to three articles on health economics during 1975 – 2022. Table 2 lists the top 10 most prolific journals, and together they have published 364 articles, which means 43% of all publications in the sample. The leading journal is the Value in Health (Impact Factor = 5.156) with 160 papers meaning 9.8% of all publications from the sample.

**Table 2:** Top 10 productive journals in health economics research

Rank	Journals	Pubs	%	Category (Quartile)	JIF
1	Value Health	160	9.87	Economics – SSCI (Q1) Health policy & services – SSCI (Q1) Health care science & service – SCIE (Q1)	5.156
2	Health Economics	50	3.08	Economics – SSCI (Q2) Health policy & services – SSCI (Q3) Health care sciences & services – SCIE (Q3)	2.395
3	British Medical Journal	39	2.40	Medicine, general & internal – SCIE (Q1)	17.215
4	Pharmacoeconomics	29	1.79	Economics – SSCI (Q1) Health policy & services – SSCI (Q1) Health care sciences & services – SCIE (Q2) Pharmacology & pharmacy – SCIE (Q2)	4.579
5	Health Policy	17	1.04	Health policy & services – SSCI (Q2) Health care sciences & services – SCIE (Q2)	3.255
6	Social Science Medicine	17	1.04	Public, environmental & occupational health – SCIE (Q2) Public, environmental & occupational health – SSCI (Q1) Social sciences, biomedical – SSCI (Q1)	5.379
7	Lancet	14	0.86	Medicine, general & internal – SCIE (Q1)	202.731
8	New England Journal of Medicine	14	0.86	Medicine, general & internal – SCIE (Q1)	176.082
9	European Journal of Public Health	12	0.74	Public, environmental & occupational health – SCIE (Q2) Public, environmental & occupational health – SSCI (Q2)	4.424
10	South African Medical Journal	12	0.74	Medicine, general & internal – SCIE (Q3)	0.566

Note: Pubs = publications, % = share in total analysed documents (1620), SSCI = Social Science Citation Index, SCIE = Science Citation Index Expanded, JIF = Journal Impact Factor according to Journal Citation Reports 2021 (calculated from data indexed in the Web of Science Core Collection), Quartile = category ranking by JIF

Source: Authors

### Co-authorship analysis

**Fig. 3:**
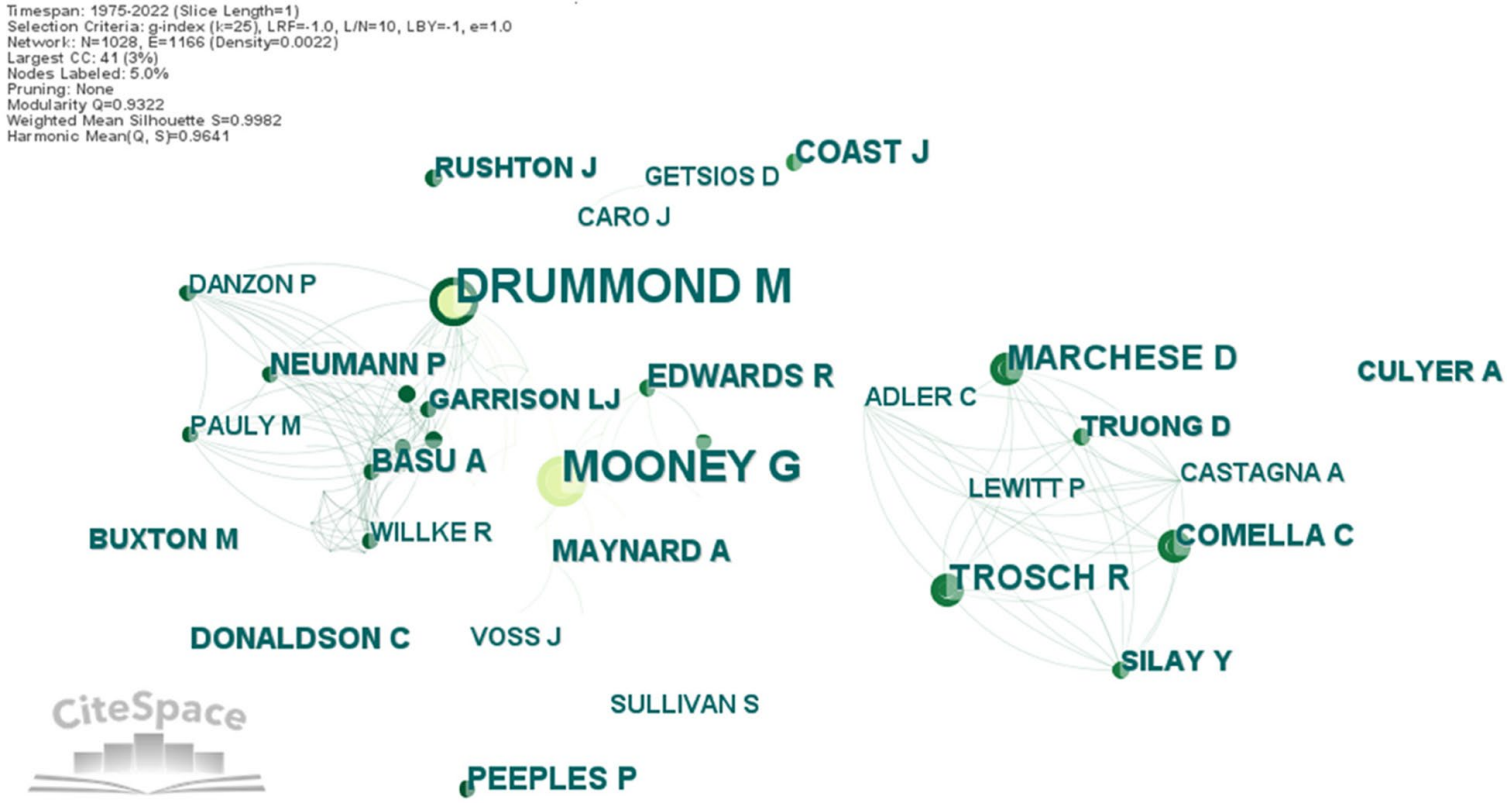
The network of authors’ collaboration in health economics. Source: Authors

Co-authorship networks and social network analysis are becoming more and more effective techniques for evaluating collaboration patterns and locating top scientists and institutions [26]. The author collaboration network can help identify authors with high contributions and reveal the co-operative relationships between the authors. By using CiteSpace, the co-authorship network was created without pruning the sliced networks. Co-authors network has 1028 nodes and 1166 links. Figure 3 presents the network between the most collaborative authors in health economics, all of them published 4 or more publications as co-authors. As indicated by the node name, each node represents a different author, and the font size corresponds to the number of publications for each author. The connections made by the co-authorship of researchers are represented by the interconnections between each pair of nodes. The degree of cooperation between the two authors is indicated by the thickness of the link.

Co-authors’ map shows that there are not strong collaboration relationships between authors, the network density level is 0.0022. Moreover, they are divided in small research groups and cooperation for research in health economics is insignificant. Top five collaborative authors are Drummond M. (20 publications), Mooney G. (16), Trosch R. (8), Marchese D. (8) and Fuchs V. (8). They are followed by Basu A. (7), Edwards R. (7), Coast J. (7), Peeples P. (7) and Comella C. (6).

In Fig. 3 it can be seen the cooperation between two research teams. These research teams are formed around key authors in health economics and integrated as most collaborative ones. First research team is created around Drummond M. and Mooney G. They published in 1982 and 1983, in British Medical Journal, 9 papers about different aspects of health economics [27, 28]. The second research team is created around Trosch R. and Marchese D., who participated between 2012 and 2015 at several annual meeting, conferences, and congresses to present their work about clinical and health economics outcomes registry in cervical dystonia [29, 30]. There are 72 scholars as co-authors in at least 3 publications showing a weak cooperation in health economics. From the perspective of citation burst, there are 5 bursting authors with a burst duration between 2 and 8 years: Drummond M. 1981–1999, Mooney G. 1982–1986, Marchese D. 2012–2015, Trosch R. 2012–2015, and Peeples P. 2018–2020. Bust analysis confirms the existence of the two research teams and their period of activity.

**Fig. 4:**
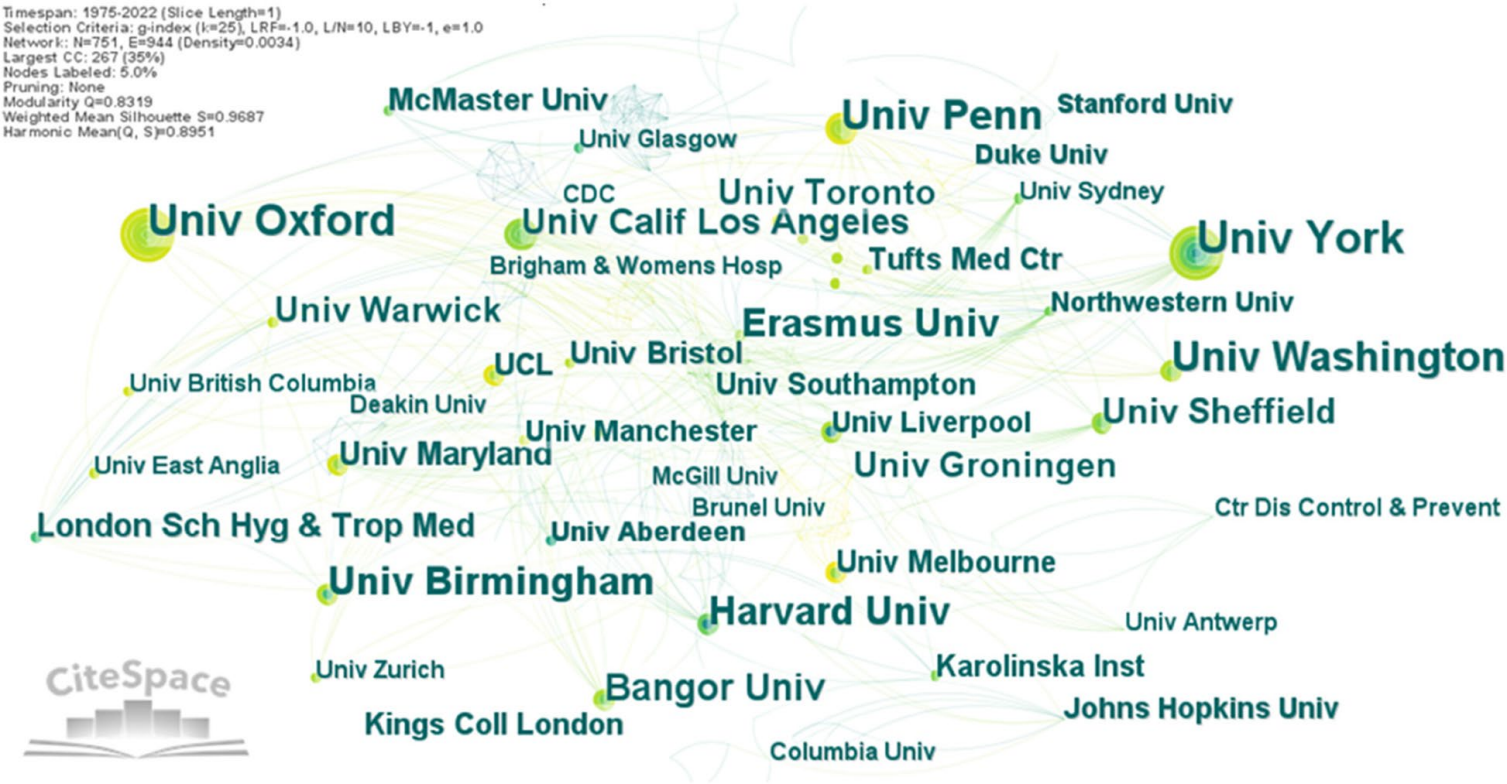
The network of institutions’ collaboration in health economics. Source: Authors

We continue exploring the co-authorship analysis by studying the level of cooperation between institutions. For this purpose, we generated a network where the nodes are the institutions, and we did not used pruning methods. The level of cooperation is revealed by the thickness between institutions’ nodes. The network contains 751 nodes, 944 links, and a density of 0.0034. In Fig. 4 are labelled the institutions with more than 4 collaborative papers, the label size is depending on the number of collaborative publications. No institution has a large value of centrality, meaning that cooperation among the analysed institutions is weak, the links are very transparent because of an insignificant number of publications written by collaboration between organizations or universities.

As seen in Fig. 4, the top-10 most collaborative institutions in health economics area are: University of York (28 publications), University of Oxford (23), University of Pennsylvania (21), University of Washington (20), University of Birmingham (17), Erasmus University (16), Harvard University (16), Bangor University (15), University of California Los Angeles (13) and University of Toronto (12). There are six institutions for which there was identified citation burst as follows: University of Oxford 2016–2020, University of Pennsylvania 2017–2022, University California Los Angeles 2013–2016, King’s College London 2006–2011, London School of Hygiene & Tropical Medicine 2008–2010, University of Washington 2015–2018. Cooperation among institutions is depending on cooperation among authors. It is understood that poor collaboration at the individual level is followed by an identical one at the organizational level.

**Fig. 5:**
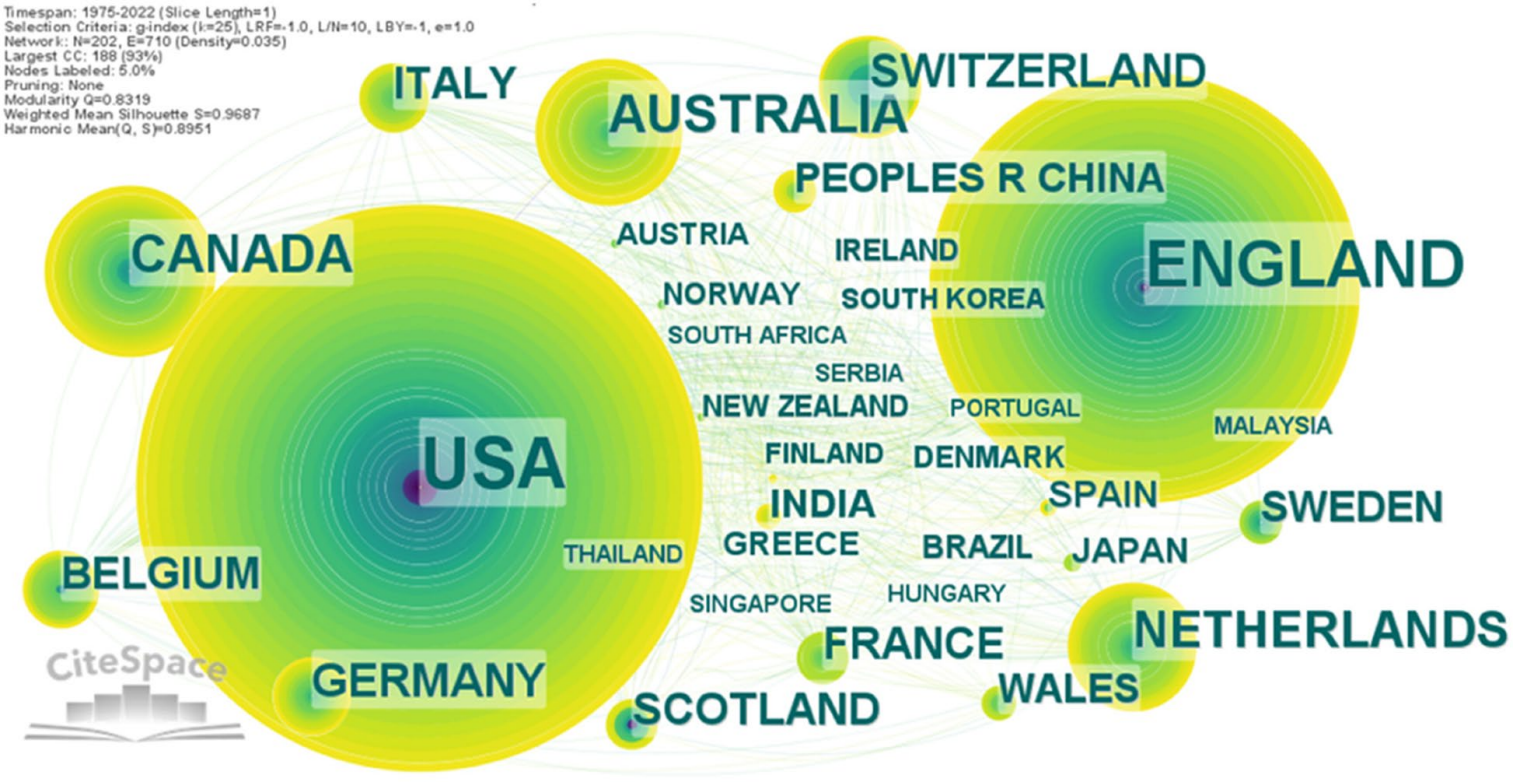
The network of countries’ collaboration in health economics. Source: Authors

Progress in any field can be achieved only by communication. Analysing country co-authorship may lead to identification of leading states in health economics research. The visualisation map for country collaboration reveals a network of 202 nodes, 710 links and 0.035 density. It should be noted that country co-authorship network has a density 10 times larger than institutions co-authorship network. The map was generated in CiteSpace without pruning parameter. In Fig. 5 are displayed the countries having more than 5 collaborative health economics-related publications.

As can be observed, the biggest nodes correspond to the most prominent and cooperative nations. The collaboration between institutions from these nations is shown by the links between the nodes. The discrepancies between the first two countries and the other states are obvious. The network of the most collaborative country, the USA, consist in 521 publications. It is followed by England with 344 publications. It is obvious that these two nations played a crucial part in worldwide academic exchanges in health economics area. The third and the fourth most collaborative countries are Canada (105 publications) and Australia (100 publications), which shows a degree of cooperation 5 times lower than that of the leading country. The top-10 most collaborative countries continue with the following nations: Netherlands (74 publications), Germany (58), Switzerland (56), Scotland (48), France (46) and Italy (43). Citation burst was identified for 4 countries: the USA 1975–1981, Scotland 1982–2003, Switzerland 1999–2006, and China 2020–2022. Citation burst analysis reveals that China, which stated to published research in health economics in 2006, faces an upward trend in the last two years.

### Co-citations analysis

**Fig. 6:**
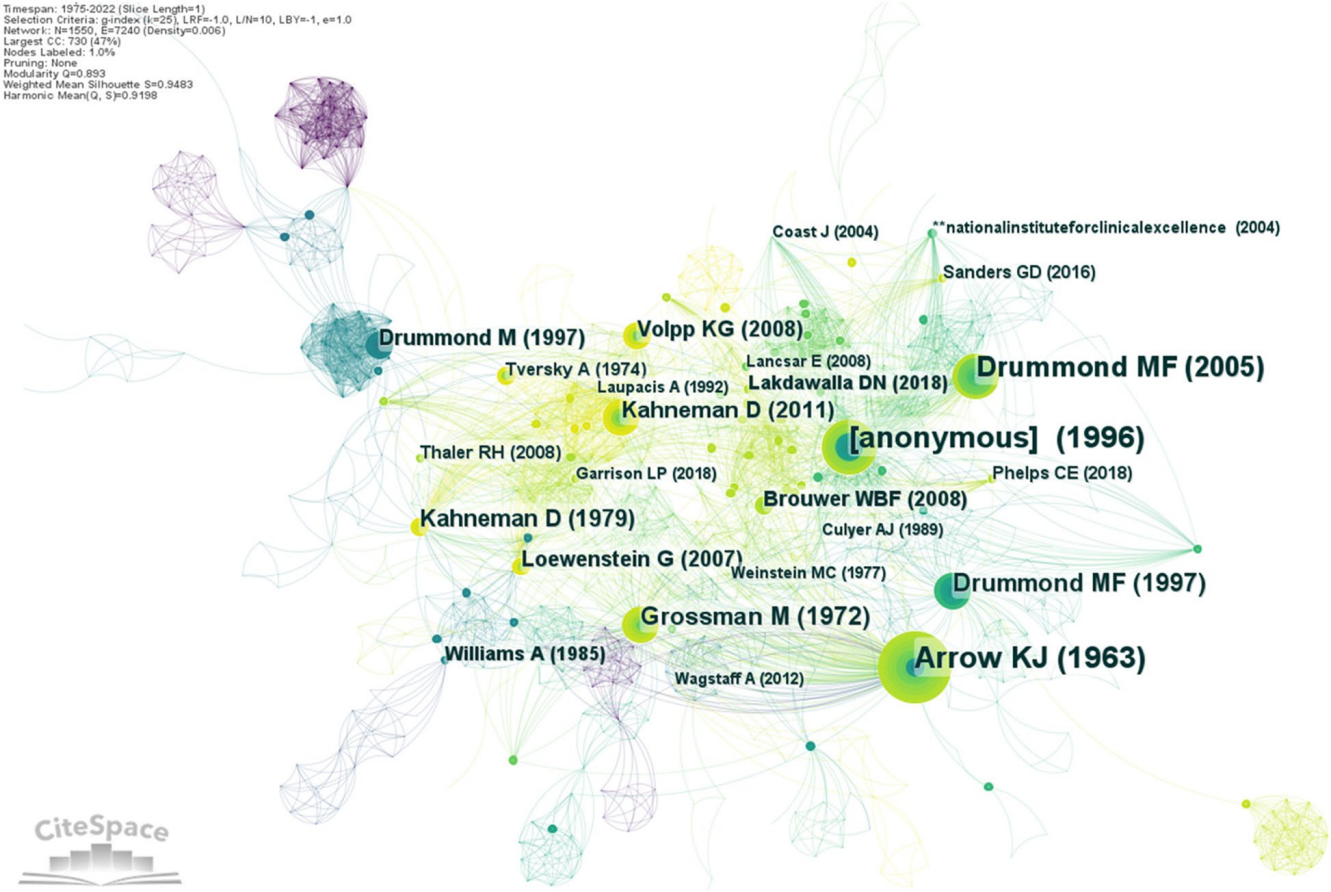
Visualization of reference co-citation networks for health economics research. Source: Authors

The following step of our current analysis is to find the most frequently cited publications in health economics sector. Co-citation reference analysis help to identification of the most important references in health economics. 16,755 references are linked to our sample. We obtain a co-citation network of 1550 nodes and 7240 links with a density of 0.0060. The network map was obtained without pruning parameter. In Fig. 6 are labelled the papers with more than 5 co-citations. Table 3 lists the top 10 articles in the field of health economics by the number of citations.

**Table 3:** Distribution of the top 10 cited references in health economics research

Rank	Reference	Journal	Year	Frequency	Burst	DOI / Link
1	Arrow K. J	American Economic Review	1963	41	9.58	https://assets.aeaweb.org/asset-server/files/9442.pdf [31]
2	[Anonymous]	Cost Effectiveness	1996	38	8.68	[32]
3	Drummond M.F	Methods for the Economic Evaluation of Health Care Programmes	1997	31	8.42 / 6.57	Book – 2^nd^ edition [33]
4	Drummond M.F	Methods for the Economic Evaluation of Health Care Programmes	2005	29	8.76	Book – 3^rd^ edition [34]
5	Grossman M	Journal of Political Economy	1972	18	4.35	10.1086/259880 [35]
6	Kahneman D	Econometrica	1979	17	4.38	10.2307/1914185 [36]
7	Kahneman D	Thinking, fast and slow	2011	14	5.03	Book – 1^st^ edition [37]
8	Loewenstein G	The Journal of the American Medical Association	2007	12	4.34	10.1001/jama.298.20.2415 [38]
9	Volpp K.G	The Journal of the American Medical Association	2008	11	3.95	10.1001/jama.2008.804 [39]
10	Brouwer W.B.F	Journal of Health Economics	2008	10	4.17	10.1016/j.jhealeco.2007.07.003 [40]

Source: Authors

As we expected, the most influential paper is published by Arrow K.J. in 1963. In his paper, the author investigates and studies the unique distinctions between medical care and other goods and services in normative economics. He focuses on medical-care industry and its efficacy by rethinking the industry from economics perspective. This publication is the basic brick in the conceptualization of health economics. Unfortunately, this part of analysis reveals some basic limitation in bibliometric analysis: incomplete and compromised database because of incorrect data filled by authors. As it can be seen in Fig. 6, the second most influential paper belongs to an anonymous author who wrote in 1996 a paper about cost effectiveness. A manual search in references database revealed the possibility to correlate the anonymous publications to a book written by Gold M.R., Siegel J.E., Russell L.B. and Weinstein M.C. The authors published in 1996 a book about cost effectiveness in health and medicine and there are several book reviews about it. The third and the fourth most co-cited publications are signed by Drummond M.F. and his co-authors. In fact, it is about a book entitled “Methods for the Economic Evaluation of Health Care Programmes”, first published in 1987 at and then renewed in the following editions: 1997 (2nd), 2005 (3rd) and 2015 (4th). Regardless the edition number, the book is a worldwide bestseller and it very cited in health economics research. It should be mentioned that the 2nd edition of the book appears twice in the database because some authors incorrectly cited Drummond. There are many book reviews for this book because it describes techniques and tools for evaluation of health care programs. It provides syntheses of new and emerging methodologies, and it is less concerned with the theoretical and ethical foundations of the methodologies (Drummond M.F et all, 2005). The book promotes basic health economic concepts and theories.

The citation burst was checked to see the period when a document citation increases sharply in frequency. There are 12 cited papers with citation burst fluctuating from 3.95 for Volpp K.G (2008) and 9.58 for Arrow K.J. (1963). Ten of twelve papers with citation burst are the ones from Table 3, the most co-cited documents in health economics. The top-10 papers by burst are Arrow K.J. 1963 (period 2012–2018, citation burst 9.58), Drummond M.F. 1997 (2000–2008, 8.76), Anonymous 1996 (1999–2011, 8.86), Drummond M.F 2005 (2008 – 2019, 8.42), Kahneman D. 2011 (2013–2022, 5.03), Williams A. 1985 (1986–1998, 4.44), Lakdawalla (2018–2022, 4.44), Kahneman D. 1979 (2019–2022, 4.38) and Grossman M. 1972 (2016–2019, 4.35).

Two of Kahneman D.’s works stands out. One of them is represented by a book, another worldwide bestseller, entitled “Thinking, Fast and Slow” published in 2011 in London. His psychological book is appreciated because it aids in the public understanding of issues related to engineering, medicine, and behavioural science. The second paper is written by Kahneman D. and Tversky A. in 1979 and presents opponents of the anticipated utility theory as a framework for risky decision-making and introduces an alternative model called prospect theory.

**Fig. 7:**
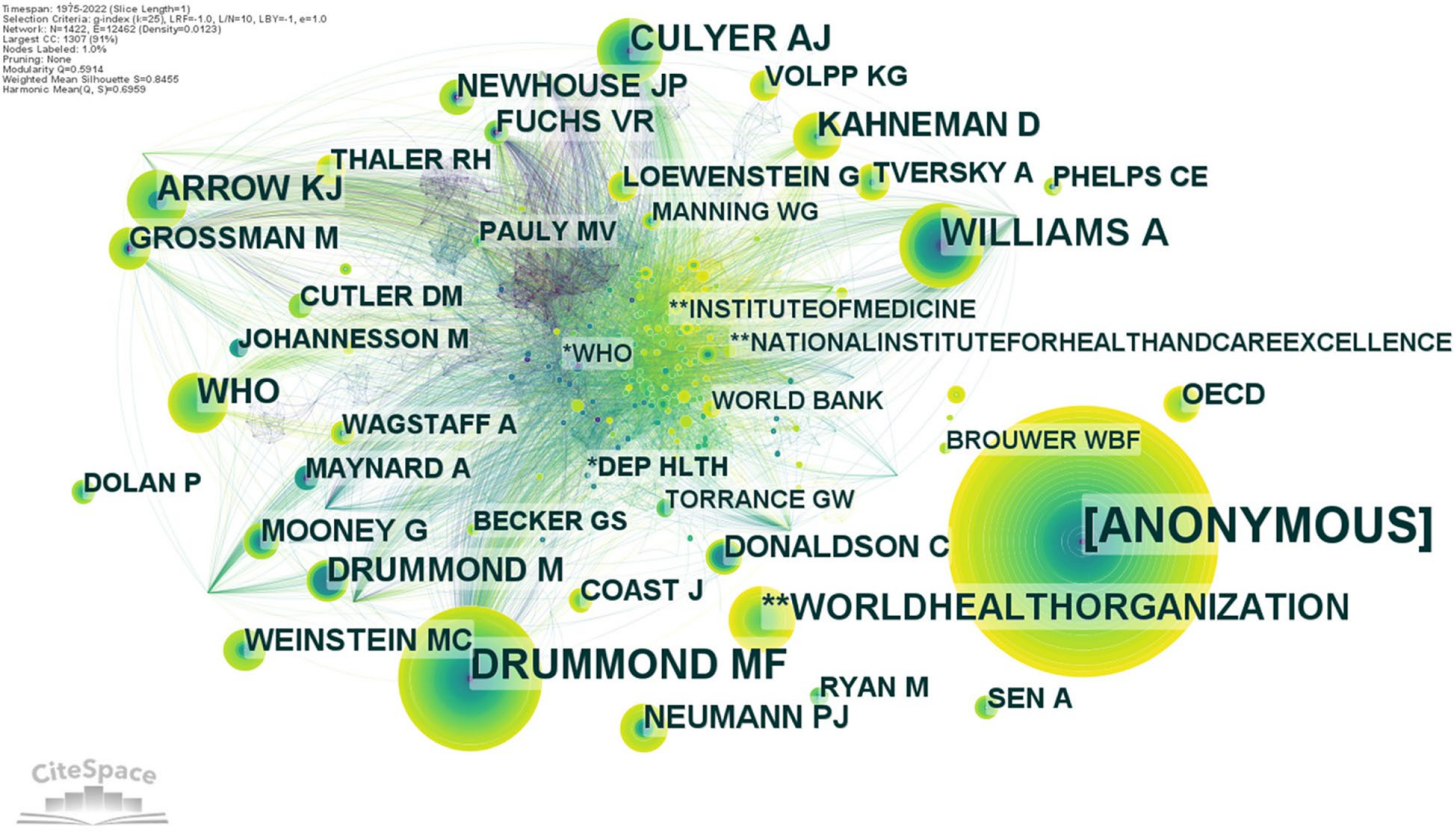
Visualization of authors co-citation networks for health economics research. Source: Authors

We can find highly cited authors whose work is well known in the health economics research community by using author co-citation networks. CiteSpace configurations are the same. The network of co-cited writers has 1422 nodes, 12,462 linkages, with a density of 0.0123. The node size reflects the number of co-citations by author. In Fig. 7 the nodes with co-citations over 14 are labelled by the corresponding first author. Once again there are incomplete data in the database. We face with an anonymous person as the most cited author in health economics research. This author without name was 300 time co-cited. We manually checked the database to find additional information about this anonymous author. According to the findings we assume it is about Margolis H. who published in 1982 a book about selfishness, altruism, and rationality. Margolis H. is a professor at the University of Chicago and in his book about social choice propose and argue a distinction between self-interest and group-interest for a person, and he also develop an equilibrium model for his theory [41].

Drummond M.F. is on the second position, positioning himself with two publications in the top-10 most co-cited authors. Once again it is about his publication with Mooney G.H. about Essentials in Health Economics which was already mentioned in the paper. Williams A. is the third co-cited author, followed by Culyer A.J and Arrow K.J. It should be noted that World Health Organization’s (WHO) publications are ones of the most co-cited document in health economics research. Unfortunately, it is hard to identify the titles of WHO’s publications from 1993 and 2009 (see Table 4) because there is more than one publication per year for this international organization. However, we assume that it is about an anonymous publication focused on tuberculosis as a worldwide problem [42] (published in 1993) and a publication about health risk at the global level [43] (published in 2009).

**Table 4:** Distribution of the top 10 cited authors in health economics research

Rank	Authors	Affiliation	Year	Frequency	Centrality	Burst	Sigma
1	Anonymous	-	1982	300	0	-	1
2	Drummond M.F	University of York—UK	1982	125	0	3.90	1
3	Williams A	University of York—UK	1982	78	0	7.63	1
4	World Health Organization	Geneva—Switzerland	1993	66	0	7.02	1
5	Culyer A.J	University of York—UK	1975	62	0	-	1
6	Arrow K.J	Stanford University—USA	1977	57	0	6.50	1
7	World Health Organization	Geneva—Switzerland	2009	47	0	8.09	1
8	Drummond M	University of York—UK	1988	44	0	9.26	1
9	Kahneman D	Princeton University—USA	2000	44	0	7.55	1
10	Newhouse J.P	Harvard University—USA	1977	39	0	-	1

Source: Authors

There are no scholars who have a betweenness centrality greater than zero. This indicates that there is no author more influential than other scholars, and no one exert a significant influence on the evolution of health economics research. The evolution of health economics theory was influenced by all the authors discussed in this paper.

In terms of burstiness, there are 35 cited authors with citation burst between 9.26 and 3.90. It means that their papers were intensively cited during a specific period. The top-10 cited authors by bursts is Drummond M. 1988 (bursts of 9.26, period 1995–1999), Maynard A, 1982 (8.60, 1998–2003), WHO 2009 (8.09, 2009–2015), OECD 2013 (7.77, 2013–2022), Williams A. 1982 (7.63, 1986–2003), Johannesson M. 1996 (7.59, 1996–2003), Kahneman D. 2000 (7.55, 2016–2022), WHO 1993 (7.02, 2011–2022), Cutler D.M. 2007 (6.97, 2012–2016) and Donaldson C. 1995 (6.94, 1995–2003). Even if they are not included in the previous ranking, the following cited authors should be mentioned because their burstiness periods exceeds 10 years: Fuchs V.R. 21 years (bursts of 4.54, period 1977–1998), Williams A. 17 years (7.63, 1986–2003), Mooney G. 14 years (5.29, 1995–2009), Dolan P. 14 years (4.84, 2003–2017) and Weinstein M.C. 13 years (4.14, 1999–2011).

**Fig. 8:**
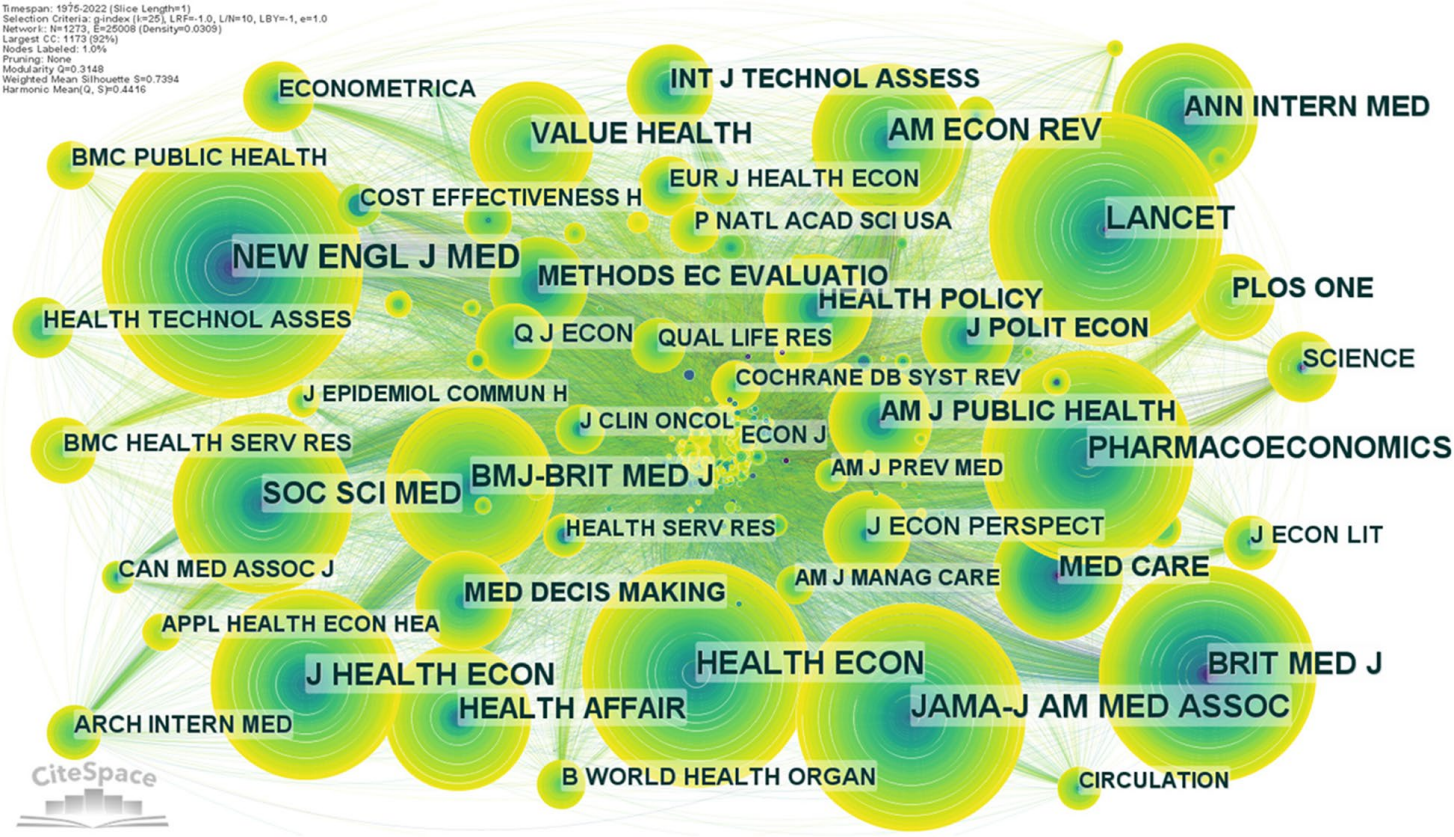
Journal co-citation network visualization for health economics research. Source: Authors

The same way as previous maps, the cited journal visualization map for health economics research (Fig. 8) was created in CiteSpace, but this network has 1273 nodes (cited journals), 25,008 linkages, and a density of 0.0309. The cited journals with more than 38 citations are labelled in the network.

The top ten journals by citations in health economics are presented in Table 5. The BMJ – British Medical Journal (381 citations) is the journal published by British Medical Association and the most prominent cited journal in health economics area. It is followed by the New England Journal of Medicine (306 citations) and The Lancet (257 citations). The journal published by American Medicinal Association ranks on the fourth place. A journal that receives a lot of citations and has a high citation burstiness score has garnered the interest of academics recently.

**Table 5:** Distribution of top 10 cited journals for health economics research

Rank	Cited journal	Frequency	Centrality	Burst	Sigma	JIF
1	British Medical Journal	381	0	20.22	1	17.215
2	The New England Journal of Medicine	306	0	8.29	1	176.082
3	The Lancet	257	0	-	1	202.731
4	The Journal of the American Medicinal Association	251	0	-	1	157.375
5	Health Economics	242	0	-	1	2.395
6	Pharmacoeconomics	211	0	8.67	1	4.579
7	Journal of Health Economics	194	0	-	1	3.804
8	American Economic Review	175	0	-	1	11.490
9	Social Science & Medicine	168	0	-	1	5.379
10	Value Health	144	0	13.15	1	5.156

*Note*: *JIF* Journal Impact Factor according to Journal Citation Reports 2021; Since 2013 British Medical Journal transformed in BMJ – British Medical Journal and its JIF is 96.216

Source: Authors

The citation surge affects 70 cited journals. The cited journal with the strongest citation bursts is Plos One (21.79, 2014–2022), which is not the most cited one. It is followed by British Medical Journal (20.22, 1982–2006), Value Health (13.15, 2018–2022), BMJ Open (12.48, 2017–2022), Applied Health Economics and Health Policy (10.38, 2017–2022), BMC Health Services Research (10.16, 2019–2022), Frontiers in Public Health (9.99, 2020–2022), Cost Effectiveness and Resource Allocation (9.66, 1998–2005), JAMA Internal Medicine (9.24, 2019–2022) and BMC Public Health (8.71, 2016–2022). It should be noted that 8 cited journals of the ranking are bursting to the present. British Medical Journal (24 years), American Journal of Psychiatry (15 years), The Journal of Health Services Research and Policy (14 years), The New England Journal of Medicine (13 years) and Medical Care (12 years) are the cited journals with the longest periods of bursting, even if the interest in these journals is currently low. It must be added that four of the most cited journals in health economics research are on a top-10 list of journals with the highest JIF in 2021. All these journals are one of the most influential journals in health research.

### Co-occurrence analysis

**Fig. 9:**
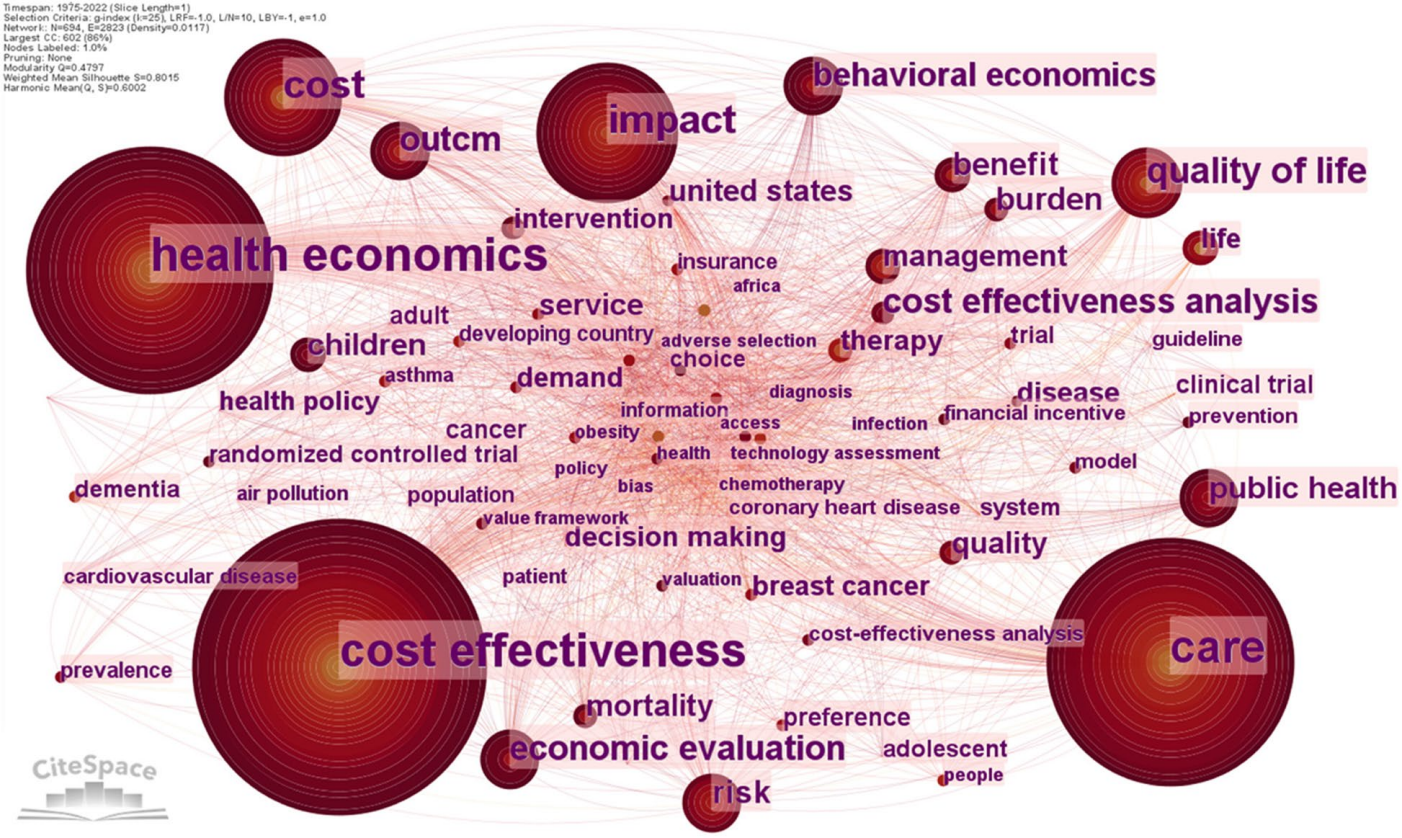
Keywords co-occurrence network for health economics research. Source: Authors

In this section of the analysis, we can pinpoint the key ideas and areas of interest in health economics research. To discover the primary study subjects in many scientific research domains, keywords are generally regarded as one of the most crucial elements of any research paper [44]. Co-occurrence analysis is used to identify the conceptual structure of the field. Without any pruning, the network of related keywords is shown in Fig. 9. The network of co-occurred keyword has 694 nodes (keywords), 2823 links (connections), and a density of 0.0117. One percent of all keywords, those with a frequency greater than or equal to five, are labelled.

Table 6 presents the top 30 keywords which are used and connected in the 1620 analysed papers. “Health economics” and “cost effectiveness” are the most co-occurred items in health economics research, they have been connected for 121 times. “Care” follows them as the second high-count keyword with a frequency of 115. One crucial statistic used in the analysis of the keyword co-occurrence network is centrality. Centrality shows a keyword's strength, influence, or other specific characteristics. In this analysis all the keywords have a null betweenness centrality.

**Table 6:** Top 30 keywords used in health economics literature

Rank	Freq	Keyword	Rank	Freq	Keyword	Rank	Freq	Keyword
1	121	Health economics	11	25	Benefit	21	15	Intervention
2	121	Cost effectiveness	12	24	Risk	22	15	Disease
3	115	Care	13	24	Burden	23	15	Decision making
4	58	Impact	14	21	Mortality	24	14	Therapy
5	58	Cost	15	21	Public health	25	13	Brest cancer
6	36	Quality of life	16	20	Children	26	13	Life
7	33	Economic evaluation	17	20	Service	27	13	Demand
8	30	Outcome	18	19	Management	28	12	Health policy
9	30	Behavioural economics	19	18	Quality	29	10	Clinical trial
10	27	Cost effectiveness analysis	20	18	United States	30	10	Cancer

Source: Authors

By using bursts detection, we tried to identify research hotspots in health economics. Surprisingly, there are only two keywords with citation bursts during 1975–2022: “behavioural economics” and “economic evaluation”. The keyword with the strongest bursts is “behavioural economics” (5.57) and it caught scholars’ attention between 2019 and 2022. The second keyword by citation bursts is “economic evaluation” (4.62). This item is bursting from 2020 to 2022. It can be observed that both research themes have short periods of bursts, and they continue bursting to present.

**Fig. 10:**
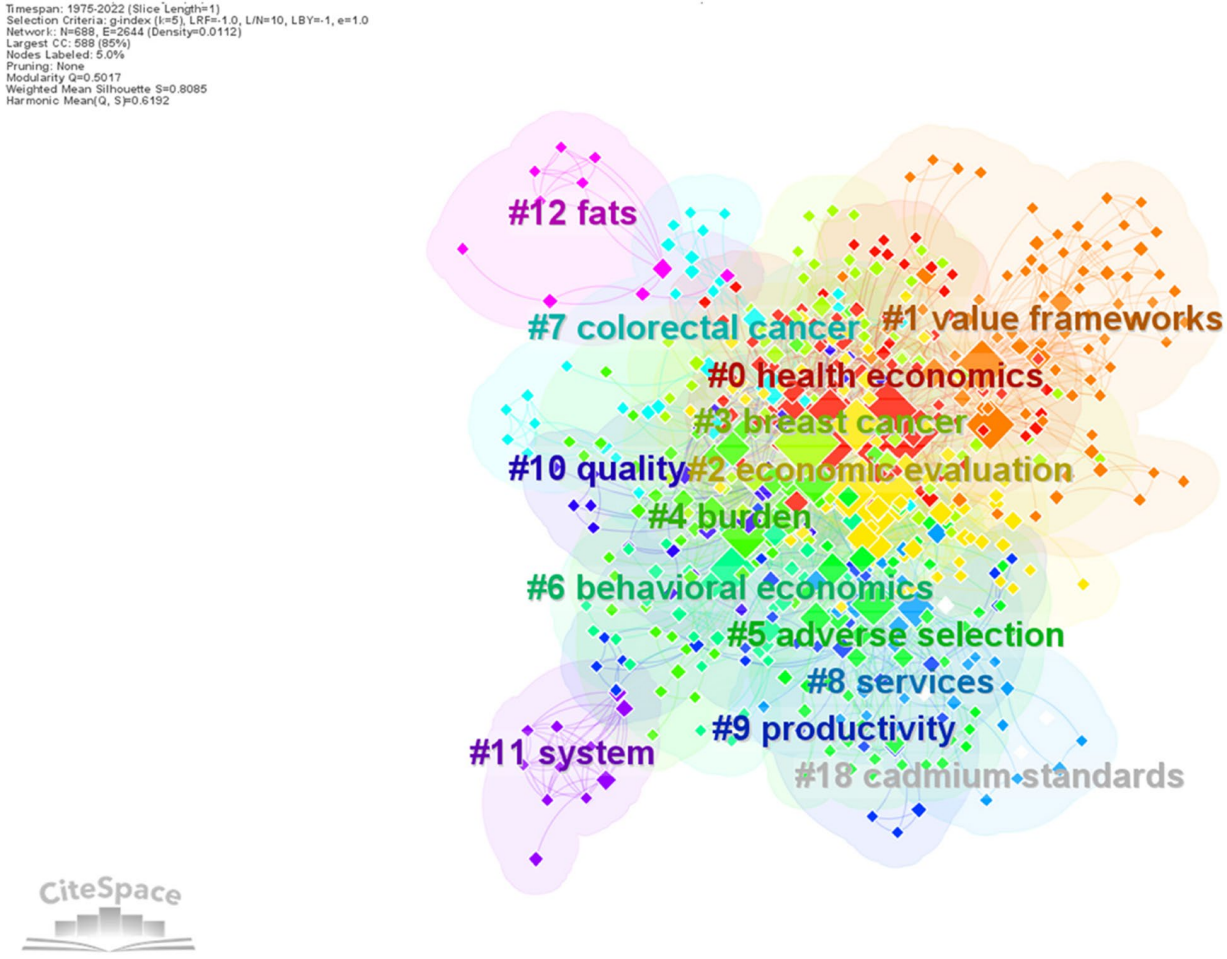
Keywords clusters. Source: Authors

CiteSpace allows a cluster analysis of keywords to identify topics that have captured the attention of researchers. By applying clustering tool, the keywords network has been divided in 14 clusters, labelled by keywords. Table 7 presents the top 10 keywords clusters, in descending order of their size, and the most used keywords in the analysed sample of publications. There are 14 clusters with different sizes, from 80 research topics in health economics to 4 research topics. Their Silhouette values varies from 0.757 to 0.995 which means that keywords match well to their own cluster. Figure 10 show that the clustering configuration is appropriate.

**Table 7:** List of top 10 keywords clusters in health economics

Cluster ID	Cluster size	Silhou-ette	Cluster label	Tag words
#0	80	0.769	Health economics	Health economics (131), cost effectiveness (85), quality of life (34), management (19), dementia (9), prevention (7), people (5)
#1	78	0.839	Value frameworks	Cost effectiveness analysis (72), health technology assessment (10), United States (8), coronary heart disease (7), surgery (4)
#2	75	0.757	Economic evaluation	Care (117), economic evaluation (34), outcome (30), benefits (21), mortality (21), disease (12), air pollution (6), asthma (6)
#3	64	0.777	Breast cancer	Impact (52), breast cancer (10), chemotherapy (5), diagnosis (5), incentives (4)
#4	60	0.772	Burden	Costs (30), risk (24), burden (23), children (20), public health (14), adults (10), adolescents (9), prevalence (8), patient (6)
#5	46	0.799	Adverse selection	Cost (22), demand (13), health policy (12), decision making (9), information (6), choice (6)
#6	35	0.784	Behavioural economics	Behavioural economics (27), financial incentives (10), randomized controlled trial (9), guidelines (7), utility (4)
#7	34	0.864	Colorectal cancer	Therapy (14), cancer (9), trial (6), association (5)
#8	33	0.794	Services	Services (17), United States (9), insurance (8), access (5)
#9	30	0.886	Productivity	Intervention (7), Africa (5), animal health (4)

The largest cluster (#0) is labelled “Health economics” and has 80 components. It contains publications about health economics, cost effectiveness, quality of life, and management. Cost effectiveness analysis and health technology assessment are subjects in the second largest cluster (#1). It is labelled “Value framework” and has 78 topics. The third cluster (#2) “Economic evaluation” contains 75 topics and the most important are care, economic evaluation, outcome, and benefits. Other research topics refer to behavioural economics, demand, cost, quality of life, risk, cancer, public heath, financial incentives, therapy, etc.

**Fig. 11:**
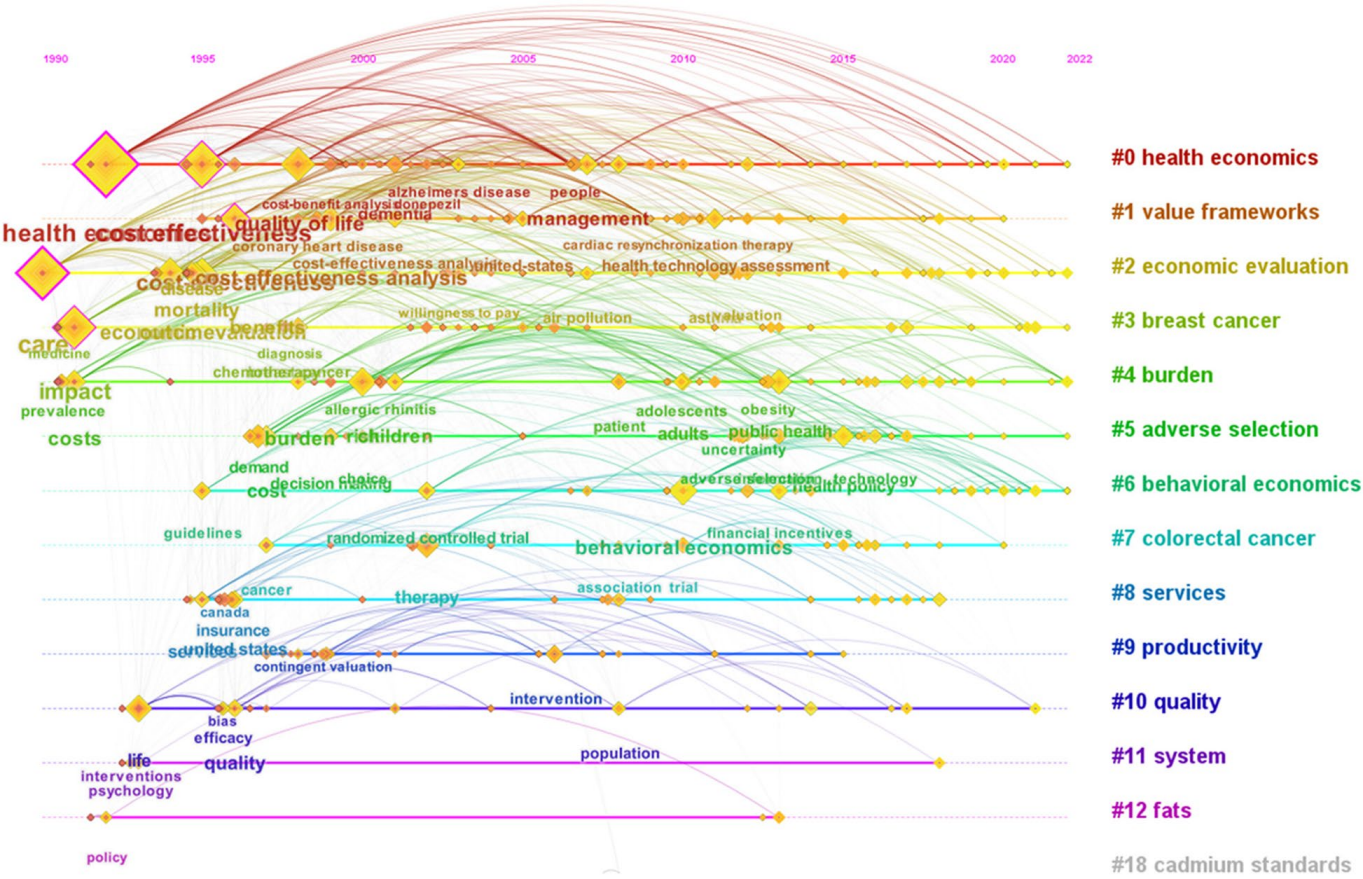
Timeline view of keywords clusters in health economics between 1990 and 2022. Source: Authors

**Fig. 12:**
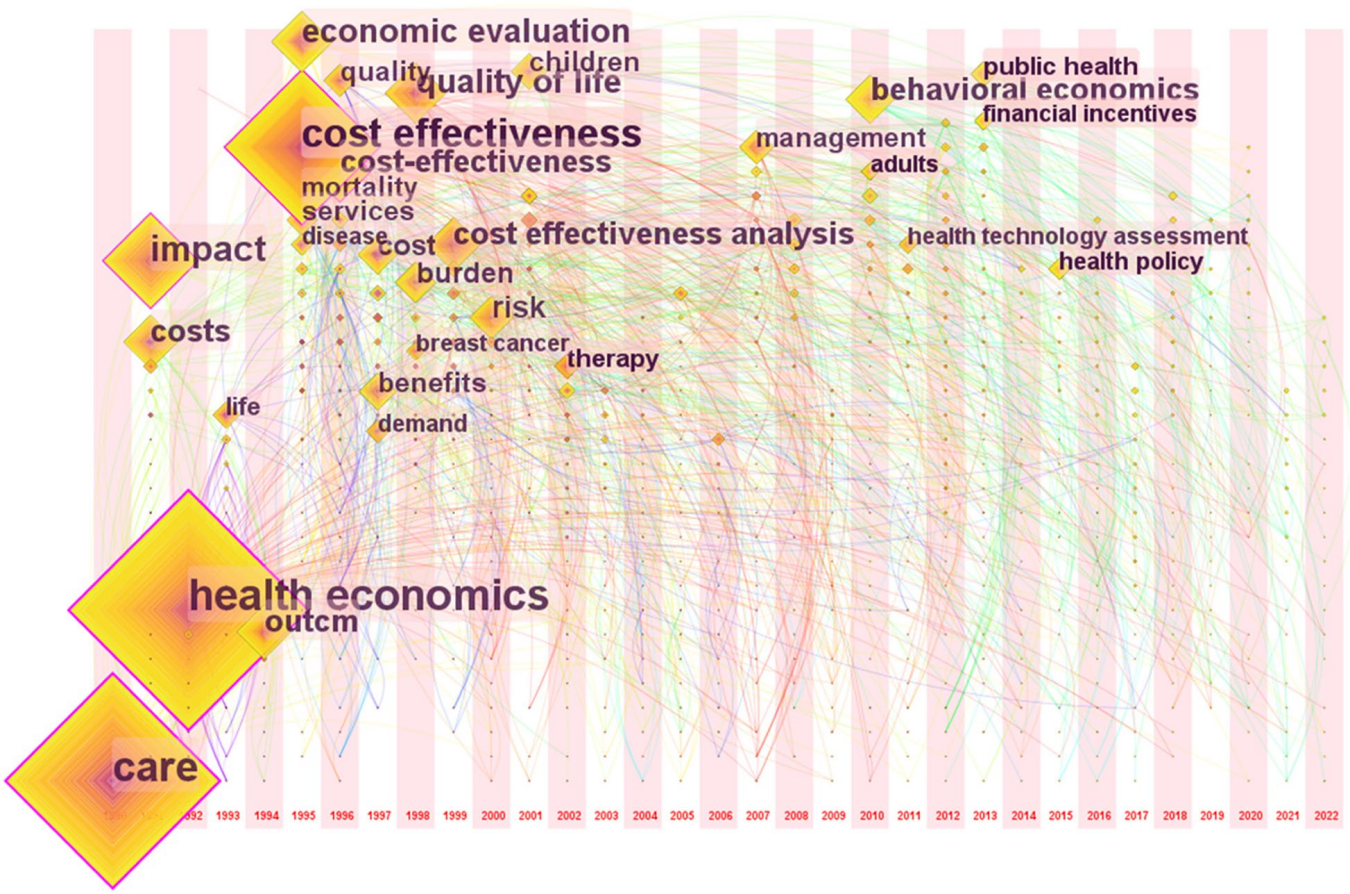
Time zone view of keywords clusters in health economics between 1990 and 2022. Source: Authors

The evolution over time of the keywords can be seen in Fig. 11, structured by cluster. CiteSpace restricts the time pane analyses to the period 1990 – 2022. Figures 11 and 12 present how interest of researchers in health economics has evolved over time. In Fig. 12 are labelled the keywords with a frequency larger than 10. In the 1990s the hot topics of research in health economics were “care”, “impact”, “health economics”, “cost”, “cost effectiveness”, “quality of life”, “outcome”, “economic evaluation”. The most debated research topics in the 2000s were “children”, “air pollution”, “patient”, “management”, “people”, “public health”, “choice”, “therapy and “risk”. In the 2010s focus is on “behavioural economics”, “population”, “obesity”, “uncertainty”, “ technology”, “health policy”, “health system”. How future research in health economics looks? It cannot be estimated with certainty, but some directions are drawn as follows: “inequality”, “care expenditure”, “health technologies”, “analysis plan”, “adaptative design”, “transparency”, “biodiversity”. These topics may shape the future literature in health economics.

The performed literature analysis enables us to respond to the research queries that were addressed in the paper's introduction, as follows:

#### How scientific production has evolved in health economics?

It can be observed a general upward trend of health economics publications, but with numerous upward and downward fluctuations, generating sinusoidal cycles with an average duration of 3–4 years. The period 1975 – 1986 is characterized by a very low number of publications. The next two decades (1987 – 2006) are characterized by a slightly increasing trend in the number of publications, with an annual average of approximately 23 publications on health economics. The following period, 2007 – 2022 is characterized by an upward evolution of the number of health economics publications, 1068 publications with an annual average of 67 articles. The evolution of the citations’ number indicates the growing interest of specialists in researching the field, especially after 2000 when a constant and galloping annual increase in citations begins. The last 5 years show a very high interest of researchers and academics in health economics research, which is justified by the existence of worldwide Covid pandemic period.

#### Who are the most important authors and publications in health economics?

In our study, 4096 different authors were identified, and they individually published between one and 16 papers. Among the most important authors in health economics are Drummond M.F., Jonsson B., Coast J., Donaldson C. and Edwards R.T. Regarding the publication’s titles, 847 distinct journals published all 1620 documents related to health economics. Value Health, Health Economics, British Medical Journal, Pharmacoeconomics and Health Policy are among journals with high interest in health economics publications.

#### What are the geographical and institutional hubs of knowledge production in health economics?

The analysed publications involved the work of authors from 82 countries. The states with significant contributions in the field of health economics are the USA, England, Canada, Australia, and Netherlands. From the point of view of affiliation, the authors belong to 1723 institutions. The institutions with a high number of publications about health economics are University of London, University of California System, University of York, Harvard University and University of Birmingham.

#### What kind of collaboration between authors, organizations, and nations are there in the field of health economics research?

There are not strong collaboration relationships between authors. They are divided in small research groups and cooperation for research in health economics is insignificant. The most collaborative authors are Drummond M., Mooney G., Trosch R., Marchese D., and Fuchs V. There are two research teams created around Drummond M. and Mooney G., on the one hand, and around Trosch R. and Marchese D., on the other hand. Cooperation among institutions is depending on cooperation among authors. It is understood that poor collaboration at the individual level is followed by an identical one at the organizational level. The most collaborative institutions in health economics area are University of York, University of Oxford, University of Pennsylvania, University of Washington, and University of Birmingham. Regarding collaboration between countries, the USA and England played a key role in worldwide academic exchanges in health economics area, followed by Canada, Australia, and Netherlands.

#### Which are the most cited authors and the most cited papers, and which are the most attractive journals for publishing research results in health economics?

The most influential paper is published by Arrow K.J. in 1963, entitled “Uncertainty and the Welfare Economics of Medical Care”. The second most influential paper belongs to an anonymous author who wrote in 1996 a paper about cost effectiveness. We assume that is a book written by Gold M.R., Siegel J.E., Russell L.B. and Weinstein M.C., entitled “Cost-Effectiveness in Health and Medicine”. The third and the fourth most cited publications are signed by Drummond M.F. and his co-authors. In fact, it is about a book entitled “Methods for the Economic Evaluation of Health Care Programmes”, first published in 1987 at and then renewed in several editions. Another influential book was written by Kahneman D., entitled “Thinking, Fast and Slow” and published in 2011. The most cited author is Margolis H., who published in 1982 a book about “Selfishness, Altruism, and Rationality”. Drummond M.F. is on the second position with the publications about “Essentials in Health Economics”. Williams A. is the third cited author, followed by Culyer A.J and Arrow K.J. It should be noted that World Health Organization’s (WHO) publications are ones of the most cited document in health economics research. The most cited journals in health economics are The BMJ – British Medical Journal, The New England Journal of Medicine, The Lancet, Journal of American Medicinal Association and Health Economics. Beside them, other very influential journals are Plos One, Value Health, BMJ Open, Applied Health Economics and Health Policy and BMC Health Services Research.

#### What are the most debated conceptual approaches in health economics?

“Health economics”, “cost effectiveness” and “care” are the most debated concepts in health economics. But the current research hotspots in health economics are “behavioural economics” and “economic evaluation”.

## Discussions and conclusions

The current bibliographic analysis was done for a specialized literature: health economics. This analysis contributes to the evaluation of the progress of the global knowledge in health economics and to the evaluation of the interest in health economics research. Moreover, the research allows the identification of the authors who contributed to the theoretical conceptualization of health economics, but also the identification of the most cited works in the field. A bibliometric analysis of the health economics research topic was produced, based on 1620 papers that were published between 1975 and 2021 and indexed in WoS. According to the tables and figures above, we have identified the important authors, publications, nations, organizations, keywords, and references.

By giving information on the current state of the art and identifying trends and research possibilities through the selection and analysis of the most pertinent publications published in the subject of health economics, the current study completes the body of existing research.

Through an extensive field mapping, the study increases the added value for the study of health economics theory. The development patterns of health economics are described by identifying trends in research production in that field and the most productive nations. The identification of top contributors’ points to possible collaborators (universities and researchers) for additional research projects. Finding the most appealing source names reveals publishing prospects for health economics-related articles. Leading thematic areas and developing research areas can be found to help academics identify research gaps in health economics.

### Limitations and future research directions

Even though the bibliometric analysis and mapping visualization on articles relevant to health economics in the current research have produced numerous fascinating results, this methodology has several drawbacks. These limitations are due to the bibliometric analysis and quality of database. A quantitative analysis reduces the influence of subjective judgments. In several parts of the analysis, we were forces to use manual search because of inadequate or incomplete data. Maybe, manual analysis is required to learn additional specifics about different aspects of health economics theory by using a systematic review analysis.

The following limitations of the current study should be considered. First, the search strategy leads to a lost in publications which do not contain the query word in the publication title. Therefore, the main findings should be interpreted in accordance with the selection strategy used in this paper. The dataset is downloaded only from WoS, maybe multi-source searching is more convincing. Publications in other languages were not analysed. For some publications the name of author was missing. Some journals change their title in time, and they appear twice as being different journals. In this analysis it was used an inhomogeneous sample due to the type of publications.

Therefore, these restrictions remain issues that need to be resolved in additional research. To sum up, our analysis cannot cover every crucial publication concerning health economics, but we believe that the results allow us to have reliable insight into the knowledge domain. This study could be carried out in the future utilizing new search criteria, time periods, or bibliometric analytic parameters.

## Data Availability

The data can be extracted from Web of Science. All data are available upon application.

## Abbreviations

EU: European Union
GDP: Gross Domestic Product
JIF: Journal Impact Factor
OECD: Organisation for Economic Co-operation and Development
SCIE: Science Citation Index Expanded
SSCI: Social Science Citation Index
UK: The United Kingdom
USA: The United States of America
WHO: World Health Organization
WoS: Web of Science

## References

[1] Mills A (1997). Leopard or chameleon? The changing character of international health economics. Tropical Med Int Health.

[2] Banta JE (1987). Sir William Petty: modern epidemiologist (1623–1687). J Commun Health..

[3] Arrow K (1963). Uncertainty and the welfare economics of medical care. American Economic Review..

[4] Newhouse JP (2001). Health Economics, International Encyclopedia of the Social & Behavioral Sciences. Pergamon.

[5] World Health Organization. Global spending on health 2020: weathering the storm. Geneva. 2020. https://www.who.int/publications/i/item/9789240017788

[6] OECD (2021). Health at a Glance 2021: OECD Indicators.

[7] European Commission. European Semester. Thematic factsheet – Health systems. https://ec.europa.eu/info/sites/default/files/file_import/european-semester_thematic-factsheet_health-systems_ro.pdf. Accessed 8 Dec 2022.

[8] Morris S, Devlin N, Parkin D, Spencer A. Economic Analysis in Healthcare, 2nd Edition, Wiley Publishing House, 2012

[9] Kernick DP (2003). Introduction to health economics for the medical practitioner. Postgrad Med J.

[10] Phelps C (2003). Health Economics.

[11] Cookson R, McDai D, Maynard A (2001). Wrong SIGN, NICE mess: is national guidance distorting allocation of resources?. BMJ.

[12] Arredondo A, Orozco E, De Icaza E (2005). Evidences on weaknesses and strengths from health financing after decentralization: lessons from Latin American countries. Int J Health Plan Manage.

[13] Zweifel P (2013). The present state of health economics: a critique and an agenda for the future. Eur J Health Econ.

[14] Mills A (2014). Reflections on the development of health economics in low- and middle-income countries. Proc Royal Soc B..

[15] Hall J (1998). The economics of public health. Austr NZ J Publ Health.

[16] Rubin RM, Chang CF (2003). A bibliometric analysis of health economics articles in the economics literature: 1991–2000. Health Econ.

[17] Wagstaff A, Culyer AJ (2012). Four decades of health economics through a bibliometric lens. J Health Econ.

[18] Moral-Munoz J, Moral-Munoz C, Pacheco Serrano A, Lucena-Anton SD, Santisteban-Espejo A (2020). Health economics: identifying leading producers, countries relative specialization and themes. Revista de Estudios Empresariales. Segunda época..

[19] Jakovljevic M, Pejcic AV (2017). Growth of global publishing output of health economics in the twenty-first century: a bibliographic insight. Front Public Health.

[20] Barth M, Haustein S, Scheidt B (2014). The life sciences in German-Chinese cooperation: an institutional-level co-publication analysis. Scientometrics.

[21] Ellegaard O, Wallin JA (2015). The bibliometric analysis of scholarly production: How great is the impact?. Scientometrics.

[22] Balaid ASS, Zibarzani M, Mohd Zaidi Abd Rozan. A Comprehensive Review of Knowledge Mapping Techniques. J Inform Syst Res Innov. 71–76 https://seminar.utmspace.edu.my/jisri/download/f1_finalpublished/pub9_comprehensive_knowledgemapping_techniques.pdf. Accessed 8 Dec 2022.

[23] Gogoi GR, Barooah PK. Knowledge Mapping, Intellectual Capital and Organizational Intelligence. Libr Philos Pract. 2021; 5910. https://digitalcommons.unl.edu/libphilprac/5910.

[24] Jafari M, Akhavan P, Bourouni A, Roozbeh HA (2009). A Framework for the selection of knowledge mapping techniques. J Knowl Manage Pract.

[25] Nada N, Kholief M, Metwally N (2009). Mobile knowledge visual e-learning toolkit. Proc 7th Int Confer Adv Mobile Comput Multimedia. ACM..

[26] Fonseca BD (2016). Co-authorship Network Analysis in Health Research: Method and Potential Use. Health Res Policy Syst..

[27] Mooney GH, Drummond MF (1982). Essentials of health economics: Part I-What is economics?. Brit Med J (Clin Research Ed)..

[28] Drummond MF, Mooney GH (1983). Essentials of health economics Part VI (concluded)-challenges for the future. Brit Med J (Clin Res ed)..

[29] Trosch et al. ANCHOR-CD (Abobotulinumtoxina Neurotoxin: Clinical & Health Economics Outcomes Registry in Cervical Dystonia): A multicenter, observational study of dysport in cervical dystonia: baseline data and interim outcomes data. Neurology. 2012;78(1). Meeting Abstract P01228.

[30] Trosch (2015). ANCHOR-CD (Abobotulinumtoxina Neurotoxin: Clinical & Health Economics Outcomes Registry In Cervical Dystonia): A multicenter, observational study of dysport in CD: baseline data & cycle one interim analysis. Muscale Nerve..

[31] Arrow K (1963). Uncertainty and the welfare economics of medical care. Am Econ Rev..

[32] Gold MR, Siegel JE, Russell LB, Weinstein MC (1996). Cost-Effectiveness in Health and Medicine.

[33] Drummond MF, O’Brien BJ, Torrance GW, Stoddart GL (1997). Methods for the Economic Evaluation of Health Care Programmes.

[34] Drummond MF, Sculpher MJ, Torrance GW, O'Brien BJ, Stoddart GL (2005). Methods for the Economic Evaluation of Health Care Programmes.

[35] Grossman M (1972). On the Concept of Health Capital and the Demand for Health. J Polit Econ.

[36] Kahneman D, Tversky A (1979). Prospect Theory: An Analysis of Decision under Risk. Econometrica.

[37] Kahneman D (2011). Thinking, Fast and Slow.

[38] Loewenstein G, Brennan T, Volpp KG (2007). Asymmetric paternalism to improve health behaviors. JAMA.

[39] Volpp KG, John LK, Troxel AB, Norton L, Fassbender J, Loewenstein G (2008). Financial incentive-based approaches for weight loss. JAMA.

[40] Brouwer WBF, Culyer AJ, van Exel NJA, Rutten FHR (2008). Welfarism vs. extra-welfarism. J Health Econ..

[41] Margolis H (1982). Selfishness, Altruism, and Rationality.

[42] Unknown author. Tuberculosis: a global emergency. World Health. 1993; 46(4):3–31 https://apps.who.int/iris/handle/10665/52639

[43] World Health Organization. Global health risks: mortality and burden of disease attributable to selected major risks. 2009. https://apps.who.int/iris/handle/10665/44203

[44] Gao F (2019). Bibliometric analysis on tendency and topics of artificial intelligence over last decade. Microsyst Technol.

